# The TFF Peptides xP1 and xP4 Appear in Distinctive Forms in the *Xenopus laevis* Gastric Mucosa: Indications for Different Protective Functions

**DOI:** 10.3390/ijms20236052

**Published:** 2019-11-30

**Authors:** René Stürmer, Jana Reising, Werner Hoffmann

**Affiliations:** Institute of Molecular Biology and Medicinal Chemistry, Otto-von-Guericke University Magdeburg, Leipziger Str. 44, 39120 Magdeburg, Germany

**Keywords:** trefoil factor, TFF1, TFF2, lectin, mucin, gastric protection, oxidative stress, thiol, cysteine, ROS

## Abstract

The gastric secretory trefoil factor family (TFF) peptides xP1 and xP4 are the *Xenopus laevis* orthologs of mammalian TFF1 and TFF2, respectively. The aim of this study was to analyze the molecular forms of xP1 and xP4 in the *X. laevis* gastric mucosa by FPLC. xP1 mainly occurred in a monomeric low-molecular-mass form and only a minor subset is associated with the mucus fraction. The occurrence of monomeric xP1 is unexpected because of its odd number of cysteine residues. Probably a conserved acidic residue flanking Cys^55^ allows monomeric secretion. Furthermore, Cys^55^ is probably post-translationally modified. For the first time, we hypothesize that the free thiol of monomeric xP1-and probably also its mammalian ortholog TFF1-could have a protective scavenger function, e.g., for reactive oxygen/nitrogen species. In contrast, xP4 mainly occurs in a high-molecular-mass form and is non-covalently bound to a mucin similarly as TFF2. In vitro binding studies with radioactively labeled porcine TFF2 even showed binding to *X. laevis* gastric mucin. Thus, xP4 is expected to bind as a lectin to an evolutionary conserved sugar epitope of the *X. laevis* ortholog of mucin MUC6 creating a tight mucus barrier. Taken together, xP1 and xP4 appear to have different gastric protective functions.

## 1. Introduction

**Figure 1:**
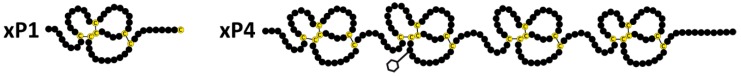
Schematic representation of the (TFF) peptides xP1 and xP4 consisting of 55 and 207 amino acids, respectively. The conserved cysteine residues including disulfide bridges are shown in yellow. The *N*-glycosylation site in xP4.1 is indicated by a hexagon, which is missing in xP4.2.

The peptides xP1 and xP4 are typical secretory products of the frog *Xenopus laevis* gastric mucosa consisting of one or four cysteine-rich trefoil factor family (TFF) domains, respectively (Figure 1) [1]. 

xP1 and xP4 belong to the family of TFF peptides, which are known for their mucosal protection and healing effects [2,3]. xP1 is synthesized mainly in gastric surface mucous cells and contains an odd number of seven cysteine residues (Figure 1) [4,5]; whereas xP4 is expressed mainly in gastric mucous neck cells, but also in esophageal goblet cells [2,5]. Because *X. laevis* is an allotetraploid species [6], two xP4 genes exist, which encode different glycosylation variants of xP4, i.e., the glycosylated form xP4.1 and the non-glycosylated form xP4.2 (Figure 1) [7]. Of note, the expression profiles of these glycosylation variants differ, xP4.2 being synthesized in the esophagus and with a decreasing gradient from the gastric fundus to the antrum [2,5,7]. In contrast, xP4.1 is synthesized in the stomach only, with a slightly increasing gradient from the fundus to the antrum [2,5,7]. Based on their structures and cellular expression patterns, xP1 is considered the *X. laevis* ortholog of mammalian TFF1; whereas xP4 appears to be the ortholog of mammalian TFF2 [2,8].

TFF1 is co-secreted together with the mucin MUC5AC from surface mucous cells and it can form heterodimers with gastrokine-2 [9,10,11]. *Tff1*-deficient (*Tff1^KO^*) mice show pleiotropic effects [12]. They obligatory develop antropyloric adenoma and about 30% progress to carcinomas [13]. Loss of *Tff1* induces a pro-inflammatory phenotype and treatment with an anti-inflammatory drug suppressed the tumor growth in these mice [14,15]. In addition, *Tff1^KO^* mice show dysregulated differentiation of pit and parietal cells in the fundic units [16] and of pit and antral gland cells in the antral units [17]. However, the molecular function of TFF1 causing this pleiotropic phenotype has not been elucidated thus far. Most notably, TFF1 dimers also have a lectin activity and bind *Helicobacter pylori* lipopolysaccharide in a pH-dependent manner [18]. Thus, TFF1 appears to play a role in mediating the tropism of *H. pylori* within the gastric mucus [19].

TFF2 is co-secreted together with the mucin MUC6 from mucous neck, antral gland, and duodenal Brunner gland cells. TFF2 strongly binds to MUC6 as a lectin, where it effects the viscoeleastic properties of gastric mucus in vitro and in vivo [20,21,22,23,24]. There are dramatic diurnal variations in the TFF2 concentrations in the gastric juice [25]. Of note, human TFF2 is *N*-glycosylated bearing the rare fucosylated LacdiNAc epitope [26,27]; whereas porcine and murine TFF2 are not glycosylated. The TFF2-binding carbohydrate epitope of MUC6 has been narrowed down to the GlcNAcα1→4Galβ1→R moiety [28]. The unusual αGlcNAc residue at the non-reducing terminals of the O-linked glycans is specifically recognized by the lectin GSA-II from *Griffonia simplicifolia* and the monoclonal antibody HIK1083. This residue is conserved in gastric gland mucins from frog to human [29,30]. Remarkably, this αGlcNAc also functions as a natural antibiotic against *H. pylori* infection [31]. *Tff2*-deficient mice (*Tff2^KO^*) show accelerated progression to *H. pylori*-induced gastritis [32], which is in line with the view that TFF2 stabilizes the gastric mucus barrier [24].

Here, we systematically investigated xP1 and xP4 from *X. laevis* gastric mucosa using size exclusion chromatography (SEC) and performed first binding studies of *X. laevis* gastric mucins with radioactively labeled porcine TFF2. These studies should mainly answer the following questions: Is xP1 associated with mucins and what are the molecular forms of xP1? Is xP4 associated with mucins as expected for an ortholog of mammalian TFF2 and do the glycoforms xP4.1 and xP4.2 behave differently? This is a further step towards understanding the molecular function(s) of xP1 and xP4, as well as of the mammalian ortholog TFF1.

## 2. Results

### 2.1. Characterzation of xP1 and xP4 in X. Laevis Gastric Extracts by SEC and Western Blot Analysis

**Figure 2:**
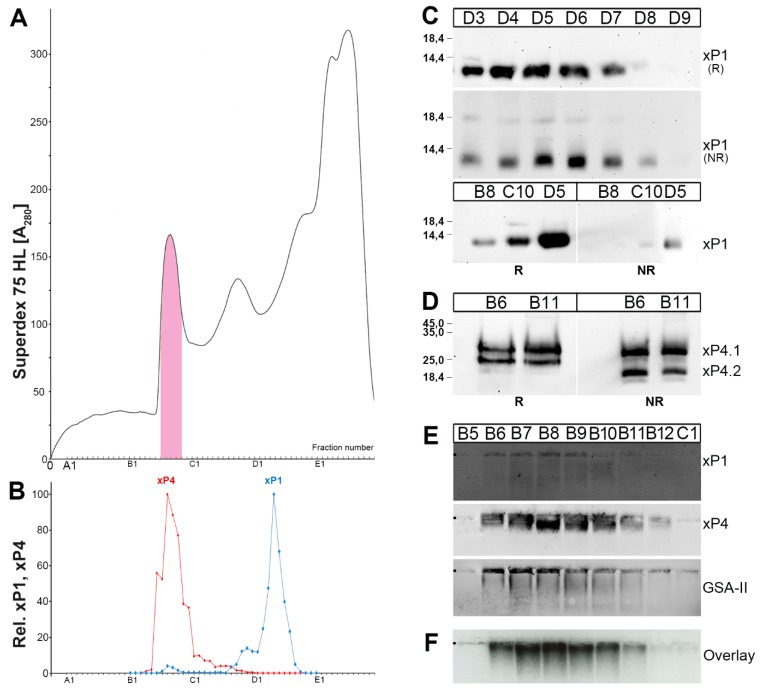
FPLC purification and analysis of xP1 and xP4 from a *X. laevis* gastric extract. (**A**) Elution profile after SEC on a Superdex 75 HL column as determined by absorbance at 280 nm (PAS-positive mucin fractions: pink). (**B**) Distribution of the relative xP1 (blue) and xP4 content (red) as determined by Western blot analysis under reducing conditions and semi-quantitative analysis of the typical 7k-and 25–30k double band intensities, respectively. (**C**) 15% SDS-PAGE and subsequent Western blot analysis of the low-molecular-mass fractions D3–D9 and the fractions B8/C10/D5, respectively. Samples were analyzed under reducing (R) and non-reducing conditions (NR), respectively, for their xP1 immunoreactivity. Molecular mass standard: left. (**D**) 15% SDS-PAGE and subsequent Western blot analysis of high-molecular-mass fractions B6/B11. Samples were analyzed under reducing (R) and non-reducing conditions (NR), respectively, for their xP4 immunoreactivity. The molecular mass standard is indicated on the left. (**E**) 1% AgGE and subsequent Western blot analysis of high-molecular-mass fractions B5–C1. Shown are reactivities for xP1, xP4, GSA-II and (**F**) the hybridization signals (autoradiography) obtained after incubating the blot with ^125^I-labeled porcine pancreatic TFF2 (overlay assay). The start is marked with a dot on the left.

When gastric extracts from *X. laevis* were subjected to SEC (Figure 2), xP1 and xP4 immunoreactivities were distributed quite differently. xP1 mainly appeared in the low-molecular-mass range (about 97%) and only a small portion was associated with the periodic acid-Schiff (PAS)-positive mucin region (about 3%; Figure 2B). In contrast, xP4 was exclusively associated with high-molecular-mass mucins (Figure 2B).

Under reducing conditions, xP1 appears as a single monomeric band with the expected M_r_ of about 7k (Figure 2C). Under non-reducing conditions, xP1 appeared as two bands, i.e., a 7k- and a weak 20k-band (Figure 2C). Of special note, xP1 immunoreactivity was drastically reduced under non-reducing conditions when compared to reducing conditions (Figure 2C).

xP4 under reducing and non-reducing conditions, respectively, always appeared as a double band (i.e., glycoforms xP4.1 and xP4.2) with a M_r_ of about 30k (Figure 2D). The xP4 immunoreactivity was not changed under non-reducing conditions (Figure 2D).

Analysis of the high-molecular-mass range revealed typical mucus staining with the lectin GSA-II in fractions B6-B11/B12 (Figure 2E). A similar pattern was obtained for xP4 and also xP1 (Figure 2E), although the latter signal was considerably weaker. 

### 2.2. Binding of ^125^I-Labeled Porcine TFF2 to X. Laevis Gastric Mucin In Vitro (Overlay Assay)

The high-molecular-mass fractions were also tested for their in vitro binding capacity using ^125^I-labeled porcine TFF2 (pTFF2) in an overlay assay (Figure 2F). Clearly, ^125^I-pTFF2 bound to similar entities as the antiserum against xP4.

## 3. Discussion

### 3.1. xP1 Mainly Occurs in An Unusual Monomeric Form: Possible Functional Implications

xP1 mainly occurs as a low-molecular-mass form with only about 3% associated with the mucin fraction (Figure 2B). This situation is remarkably similar to that of the human ortholog TFF1 [11]. Based on the results from the non-reducing SDS-PAGE, the major form of xP1 is a monomer (7k-band; Figure 2C). Furthermore, a weak 20k-band could be observed, which probably represents a xP1-homodimer (similar size as described for TFF3; [33]). Alternatively, the 20k-band might represent a xP1 heterodimer with a yet unknown partner. This would be analogous to TFF1, which can form a 25k heterodimer with gastrokine-2 (GKN2) [10,11].

The occurrence of a xP1 monomer is unusual because the oxidation machinery of the endoplasmic reticulum enforces disulfide bond formation in secretory proteins [34]. Thus, xP1 containing an odd number of cysteine residues should form either a disulfide-linked homodimer or a heterodimer, such as TFF1-GKN2 [10,11]. Generally, exposed thiols act as intracellular retention signals for unassembled secretory proteins [35]. However, there are examples known where proteins are secreted despite the presence of an unpaired cysteine residue, e.g., such as Ig light chains [35]. In this case, a flanking acidic amino acid residue (aspartic acid) was shown to mask the retention signal allowing transport to the Golgi [35]. Such a case might also occur in xP1 where the C-terminal Cys^55^, expected to be the unpaired cysteine residue (Figure 1), is directly flanked by a glutamic acid residue [4]. Of note, an additional gene exists in *X. laevis* encoding a xP1 homolog, which is expressed during the larval stages and in tadpoles only, but not in the adult [36]. The corresponding peptide has been designated as xP1-L and it contains even two glutamic acid residues before the 7th cysteine residue [36]. Furthermore, mouse and human TFF1 contain even three glutamic acid residues upstream of the 7th cysteine residue (and in human TFF1 this 7th cysteine residue is flanked on top by a downstream glutamic acid residue). Such flanking amino acids are known to change the pKa of cysteine residues [37,38,39]. This might indicate that there was obviously an evolutionary pressure to change the pK_a_ of this highly conserved cysteine residue and this residue probably plays a key role for the function of TFF1.

In addition, such an unpaired cysteine residue might have also a transient function as a chaperon for the correct folding of other cysteine-rich proteins secreted by the same cells: in particular, neutral mucins are characteristic secretory products of surface mucous cells, similar to mammalian MUC5AC [5]. This hypothesis would be in agreement with the observation that in antropyloric tumors of *Tff1^KO^* mice the unfolded protein response is activated [40].

The extremely reduced immunoreactivity of xP1 against the antiserum anti-xP1-1 (generated against the very C-terminal of xP1; [5]) under non-reducing conditions (Figure 2C) might be an indication for a post-translational modification (PTM) of Cys^55^. There is an increasing number of PTMs known for cysteine residues including sulfenic and sulfinic acids [39]. Generally, thiol groups of cysteine residues are particularly susceptible to oxidation by reactive oxygen/nitrogen (ROS/RNS) species [39]. Consequently, xP1 - and its mammalian ortholog TFF1 - could have a protective scavenger function, e.g., for extracellular ROS/RNS, via their C-terminal cysteine residues. The apical surface of gastric epithelial cells is well known to release extracellular ROS by dual oxidase (DUOX) in particular during bacterial infections and chronic inflammatory diseases [41]. The generation of H_2_O_2_ by the DUOX enzyme restricts microbial colonization [41]. As a consequence, the extracelluar compartment is subject to great oxidative stress [42] and effective protection is essential for the sensitive gastric mucosa [43]. TFF1, maybe in concert with a secreted form of peroxiredoxins, could be part of a protective shield preventing inflammation triggered by ROS. This might also explain, why in mammals TFF1 is ectopically expressed during various inflammatory conditions, such as duodenal ulcers, Crohn’s disease, pancreatitis, asthma, encephalitis, and in the murine spleen after *Toxoplasma gondii* infection [44,45,46,47,48,49]. Here, TFF1 could protect from extracellular damages due to the oxidative burst, which is generated, e.g., from activated neutrophiles.

In addition, xP1 could also act as an antimicrobial peptide, maybe after reduction of disulfide bridges. Such a case was reported for human β-defensin 1 [50].

### 3.2. xP4 is Mucin-Associated: Interaction with the Ortholog of MUC6

Here, we show that xP4 is indeed bound to mucin and is comparable with TFF2 from human and pig [11,51,52]. Thus, xP4 can now be considered as the functional ortholog of mammalian TFF2 in spite of a different number of TFF domains (4 versus 2). In particular, the four TFF domains of xP4 would be perfectly designed to cross-link mucins. Both glycoforms of xP4, i.e., xP4.1 and xP4.2, are mucin-bound and completely released by boiling in SDS indicating a non-covalent binding of both xP4 forms to a mucin, which has to be considered as the ortholog of mammalian MUC6. Indeed, such a mucin has been identified in *X. tropicalis* [53]. Generally, MUC6 is present early in vertebrates, but was lost in teleost fishes [53]. Of special note, glycosylation of xP4 does not appear to influence the lectin binding to gastric mucin. First analyses of xP4 with lectins did not show any indication that the glycosylated variant xP4.1 bears the fucosylated LacdiNAc epitope (data not shown) as found in human TFF2 [27]. 

Generally, a protective function can be expected for xP4 as described for mammalian TFF2 [24], i.e., lectin binding and possibly cross-linking of the *X. laevis* ortholog of MUC6. This mucin probably bears the characteristic peripheral GlcNAcα1→4Galβ1→R moiety because porcine TFF2 is bound in vitro (Figure 2F). It is synthesized together with xP4 in mucous neck cells, and is recognized by the lectin GSA-II (Figure 2E) and the antibody HIK1083 [30]. Thus, lectin interaction of TFF2 with MUC6 seems to be an evolutionary highly conserved principle, which started already early in vertebrates [53] and even allows binding of porcine TFF2 to *X. laevis* gastric mucin (Figure 2F). A key step for this mechanism is the synthesis of the evolutionary conserved, unusual peripheral glycan αGlcNAc by α1,4-*N*-acetylglucosaminyltransferase (α4GnT); mice lacking this enzyme spontaneously develop adenocarcinoma in the gastric antrum [54].

## 4. Materials and Methods 

### 4.1. Extraction of Proteins and Purification by SEC

Proteins were extracted from the stomach (1.6–1.8 g) of *X. laevis* (purchased from the W. de Rover, Herpetological Institute, Turnhout, Belgium) with a 5-fold amount (*w*/*v*) of buffer (30 mM NaCl, 20 mM Tris-HCl pH 7.0 plus protease inhibitors) in a Precellys^®^24 lyser/homogenizer analogous as described previously in detail [27]. 

Then, 8 mL of gastric extracts were fractioned by SEC with the ÄKTA^TM^ FPLC system (Amersham Biosciences, Freiburg, Germany) as described (fraction numbering: A1-A12, B1-B12, etc.) [51]. The following column was used: HiLoad 16/600 Superdex 75 prep grade (S75HL, GE Healthcare Biosciences AB, Uppsala, Sweden; 20 mM Tris-HCl pH 7.0, 30 mM NaCl plus protease inhibitors; flow rate: 1.0 mL/min; 2.0 mL fractions).

### 4.2. SDS-PAGE, Agarose Gel Electrophoresis, and Western Blot Analysis

Non-denaturing agarose gel electrophoresis (AgGE; containing 0.1% SDS), denaturing SDS-PAGE under reducing or non-reducing conditions, and periodic acid-Schiff (PAS) staining for mucins (dot blot) were described previously [33,51].

Western blot analyses after SDS-PAGE (electrophoretic transfer) or AgGE (capillary blot) was as reported [52]. All gels after non-reducing SDS-PAGE were subjected to post-in-gel reduction with 1% mercaptoethanol at 50 °C for 5 min before blotting as described previously [51]. Gels after AgGE were directly blotted and for the detection with antisera, the proteins were additionally reduced on the membranes in situ with 1% mercaptoethanol at room temperature for 5 min.

Mucins were detected with the biotinylated lectin GSA-II from *G. simplicifolia* (2 µg/mL) as reported [51]. xP1 was analyzed with the polyclonal antiserum anti-xP1-1 (1:5000 dilution) against the C-terminal synthetic peptide FYPRATPEC as described previously [5]. Production of a polyclonal antiserum anti-xP4-1 against the C-terminal of xP4 (synthetic peptide CFYPDIEDVTIIE) was reported previously [1]. The antiserum anti-xP4-1 was used in a 1:5000 dilution. Bands were visualized with the enhanced chemiluminescence (ECL) detection system (using a secondary antibody coupled to horseradish peroxidase and luminol/p-Coumaric acid/H_2_O_2_) and the signals were recorded with the GeneGnome system (Syngene, Cambridge, UK). For semi-quantitative analysis, the relative intensity for each band within a given frame was calculated using the GeneTools gel analysis software (Syngene, Cambridge, UK) setting the highest intensity in a series to 100%.

### 4.3. TFF2 Binding Studies

TFF2 from porcine pancreas (pTFF2) was kindly provided by L. Thim (Novo Nordisk A/S, Maaloev, Denmark) [55]. Labeling of pTFF2 with ^125^I (iodogen method) and overlay assays with ^125^I-labeled TFF2 were as described in detail previously [52]. In brief, mucin containing fractions after SEC were separated by AgGE, blotted onto nitrocellulose membranes, hybridized with ^125^I-labeled pTFF2 (in 20 mM Tris-HCl pH 7.0, 2.5 mM CaCl_2_, 500 mM NaCl), and exposed to a film (autoradiography). 

## 5. Conclusions

Taken together, xP1 and xP4 probably have quite different protective functions for the gastric mucosa, which is exposed to extremely harsh conditions (e.g., hydrochloric acid as well as exogenous pathogens from the diet). For the first time, we hypothesize that the free thiol of monomeric xP1―and probably also its mammalian ortholog TFF1―might act as a scavenger for extracellular ROS/RNS. This could open interesting clinical perspectives because TFF1 has therapeutic potential, e.g., by reducing mucositis in cancer patients receiving chemotherapy [56]. For example, it could be tested whether oral application of short synthetic peptides mimicking the C-terminal end of xP1/TFF1 prevents formation of adenoma and carcinogenesis in *Tff1^KO^* mice. In addition, xP1 could possess an antimicrobial activity and have a chaperon function for the secretion of the neutral mucin from surface mucous cells. In contrast, xP4 is non-covalently bound to the *X. laevis* ortholog of MUC6 and could perfectly cross-link this mucin creating a tight mucus barrier.

## Abbreviations

AgGE	Agarose gel electrophoresis
PAS	Periodic acid-Schiff
SDS-PAGE	Sodium dodecyl sulfate-polyacrylamide gel electrophoresis
SEC	Size exclusion chromatography
TFF	Trefoil factor family
